# Maraviroc attenuates orbital remodeling, inflammation, and lipid dysregulation in a murine model of thyroid eye disease associated with Graves’ disease

**DOI:** 10.3389/fendo.2026.1717212

**Published:** 2026-02-27

**Authors:** Fahimeh Hashemi Arani, Anne Gulbins, Mareike Horstmann, Insa Nolte, Anke Daser, Nikolaos E. Bechrakis, J Paul Banga, Erich Gulbins, Anja Eckstein, Gina-Eva Görtz

**Affiliations:** 1 Department of Ophthalmology, Orbital Diseases Science Lab (ODSL), University Hospital Essen, University of Duisburg-Essen, Essen, Germany; 2 Department of Oto-Rhino-Laryngology, Head and Neck Surgery, University Hospital Essen, University of Duisburg-Essen, Essen, Germany; 3 Institute of Molecular Biology, University Hospital Essen, University of Duisburg-Essen, Essen, Germany

**Keywords:** CCR5/CCL5, inflammation, maraviroc, orbital remodeling, TED

## Abstract

**Background:**

Graves’ disease (GD) is an autoimmune condition that can extend beyond the thyroid, leading to thyroid eye disease (TED), a disorder marked by orbital inflammation and tissue remodeling.

**Methods:**

We explored the therapeutic potential of maraviroc, a CCR5 antagonist, in a mouse model of TED triggered by immunization with the human TSH receptor (hTSHR) A-subunit. Mice received pTriEx1.1neo-hTSHR A-subunit plasmid immunizations, and a subset were treated with maraviroc via drinking water. We assessed thyroid function, orbital tissue changes, immune cell infiltration, and lipid metabolism through serological testing, histology, immunohistochemistry, and untargeted lipidomics.

**Results:**

Maraviroc did not significantly affect anti-TSHR antibody production nor the degree of hyperthyroidism, though it modestly improved thyroid histopathology. Notably, it reduced key signs of orbital disease, including brown adipose tissue expansion, CCL5-positive immune cell infiltration, CD4^+^ T-cell infiltration and the presence of F4/80^+^ macrophages. Lipidomic profiling revealed distinct metabolic changes in treated mice, with reduced triacylglycerols and elevated carnitines, indicative of enhanced fatty acid utilization. Composite Z-score analysis reinforced maraviroc’s beneficial effects on orbital inflammation and remodeling.

**Conclusion:**

Maraviroc shows promise as a targeted therapy for TED in the context of GD, offering anti-inflammatory and anti-adipogenic benefits while sparing thyroid autoimmunity. These preclinical findings support further clinical investigation into its role in managing TED.

## 1 Introduction

Graves’ disease (GD) is an autoimmune disorder of the thyroid characterized by the presence of autoantibodies targeting the thyrotropin receptor (TSHR). Autoantibodies are responsible for a considerable number of the clinical manifestations of GD and serve as specific biomarkers of this autoimmune thyroid disease (1–3).

In autoimmune thyroid disease, the immune response is primarily localized to the thyroid gland, where key thyroid antigen, the TSHR is expressed. However, secondary soft tissues including eyes can also be affected because they contain TSHR that bind to these antibodies (Abs), a condition known as thyroid eye disease (TED). The strong clinical relationship between GD and TED indicates that the immunoreactivity against the TSHR occurring in both the thyroid and orbit underlies both conditions (4). TED is the most prevalent extra-thyroidal manifestation of GD, affecting around 30% of patients (5, 6). It is known that several factors increase the risk of developing and progressing TED such as smoking, elevated thyrotropin receptor antibody (TRAb) levels, radioiodine treatment and prolonged period of untreated hyperthyroidism (7–9).

A number of studies have demonstrated the expression of the TSHR is higher in the orbital fat of patients with TED than in normal orbital adipose tissues (10, 11). Thyroid stimulating immunoglobulin (TSAbs) activity on thyrocytes induces hyperthyroidism, while the activity of TSAbs on TSHR expressing orbital and/or pretibial fibroblasts provoke the release of a number of proinflammatory cytokines and antigen-specific T-cell responses, leading to systemic inflammation (7, 12, 13). This leads to the accumulation of mucopolysaccharides/glycosaminoglycans, which trap water and cause oedema (7–9, 14). Subsequently, fibrosis becomes a prominent feature. The activation of fibroblasts is caused by proinflammatory cytokines derived from locally infiltrating T cells (15) and macrophages (16).

Orbital fibroblasts (OFs) have an intrinsic pro-adipogenic phenotype and are central to the pathogenesis of TED. They express both TSHR and the insulin-like growth factor-1 receptor (IGF-1R) (14), and TSAbs activate these receptors through crosstalk (17). This activation promotes hyaluronan production, whose hydrophilic properties drive mucopolysaccharides accumulation and edema formation in orbital tissues (18, 19).

Multiple immunizations with a plasmid encoding the human TSHR A-subunit followed by electroporation are the basis for an induced mouse model of GD and TED (20–23). Previous research has shown that the mouse model was robust to variable environmental exposures and accurately reflects characteristic and stable features of TED pathology similar to those seen in individual patients (24, 25). We have shown that linsitinib (OSI-906), a potent, oral inhibitor of IGF-1R and the insulin receptor (IR), effectively blocked the development and progression of GD and TED in a preclinical mouse model. Treatment after disease onset markedly reduced disease severity and orbital inflammation (14).

In addition, our recent work demonstrated that linsitinib also prevents activation of bone marrow in experimental GD and TED (15). Linsitinib exerts immunomodulatory effects in the bone marrow by upregulation of arginase-1, an enzyme that depletes L-arginine, thereby limiting T-cell activation, which depends on sufficient arginine (22, 26). Bone marrow activation upon immunization is associated with reduced IL-10 and PGE2 levels, promoting autoimmune responses. Linsitinib reversed these changes by increasing arginase-1 expression, reduce in arginine availability, and restoring IL-10 and PGE2, collectively damping autoimmune activation (14). In addition, the C-C motif chemokine receptor 5 (CCR5), a receptor on hematopoietic stem cells (HSCs), is activated by binding of the corresponding ligand (CCL5), which is expressed y by activated T-cells, and triggers an increased myelopoiesis by HSCs. The CCR5-CCL5 axis appears to be involved in these activation steps, as demonstrated in a multiple sclerosis murine model (27). It should be noted that CCL5 has been shown to be increased in the blood plasma of TED mice but is normalized upon linsitinib treatment (14).

In the present study, we investigated the effect of maraviroc, an antagonist of the CCR5 receptor, on the active state of orbital inflammation in our experimental GD/TED mouse model. Herein, we show a positive effect of maraviroc on the outcome of experimental TED, especially on adipogenesis. These results are important in assessing potential of maraviroc for translation, as it shows therapeutic potential for treating the predominant manifestations of adipogenesis and inflammation in TED.

## 2 Methods

### 2.1 Construction of pTRiEx1.1 neo‐h TSHR A‐subunit plasmid

The expression plasmids pTriEx-1.1 neo-hTSHR A-subunit and pTriEx-1.1 neo-β-Gal were constructed and purified as previously described by Zhao et al. (23, 28).

### 2.2 Mice

Female BALB/c inbred mice, 6 weeks of age, were obtained from Envigo Netherlands GmbH and housed under specific conditions as previously described (29). All animal procedures were reviewed and approved by the State Agency for Consumer Protection and Food of North Rhine-Westphalia (LAVE), Germany. All experimental work was performed in accordance with FELASA regulations and reported following Animal Research: Reporting in Vivo Experiments (ARRIVE) guidelines.

Animals were randomly assigned to different experimental groups before the initiation of the study to ensure equal distribution across groups. During the *in vivo* studies, the burden on the mice was monitored daily using a scoring system that included body weight, general condition changes, and behavioral changes. Indeed, the burden was evidenced by weight reduction and behavioral changes. Therefore, a weight loss of 10-19% or more, accompanied by lethargy, was considered the endpoint of the analysis, and the respective animal was immediately euthanized using CO_2_ asphyxiation. Mice were euthanized by controlled exposure to 100% CO_2_ in their home cage at a flow rate of ~1.5 L/min (≈20% cage volume exchange), following the AVMA Guidelines for the Euthanasia of Animals: 2013 Edition (30). Death was confirmed by the absence of heartbeat, respiration, and corneal reflex. Data collection was performed by investigators that were blinded to the experimental conditions.

### 2.3 Immunizations and maraviroc treatment

**Figure 1:**
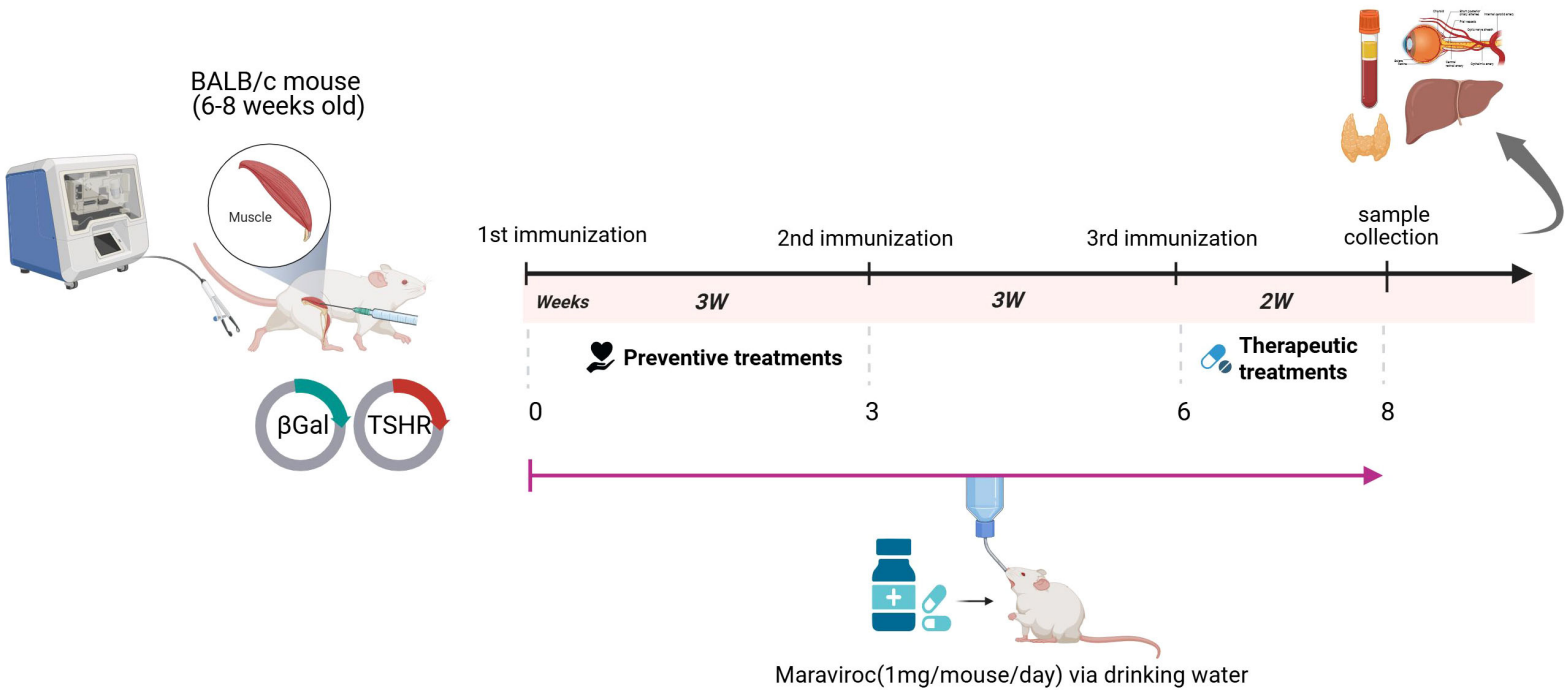
Experimental design of maraviroc treatment in a mouse model of thyroid eye disease. To induce autoimmune hyperthyroidism and the associated thyroid eye disease, six-week-old female BALB/c mice were immunized three times with a plasmid encoding the TSHR A-subunit at three-week intervals. Beginning with the first immunization, TSHR-immunized mice were treated either with maraviroc to evaluate its effect on disease progression, or with placebo. As healthy controls, a separate group of female BALB/c mice was immunized with a β-galactosidase-encoding plasmid (β-Gal). The experiment was terminated two weeks after the final immunization, and multiple analyses were performed across the different mouse groups, as illustrated in the figure.

Three groups of 12 animals each were immunized with either the eukaryotic expression plasmid pTriEx1.1neo-human (h) TSHR A-subunit or the control pTri1Ex1.1neo-ß-Gal plasmid (control ß-Gal group) as outlined in previous studies (14, 29). All mice received three immunizations spaced three weeks apart and were euthanized two weeks after the final immunization. One group within the TED category received maraviroc (Celsentri, ViiV Healthcare, United Kingdom) *ad libitum* in their drinking water from the initial immunization until the end of the experiment (a total of eight weeks) at a concentration of 300 mg/L, corresponding to an approximate dose of 1 mg per mouse/day. All groups were sacrificed eight weeks after the start of the experiment, which coincides with two weeks after the last immunization. The experimental timelines for maraviroc treatment are shown in **Figure 1**.

### 2.4 Serological analyses

Total anti-TSHR antibodies, stimulating anti-TSHR antibodies, fT3 and fT4 levels and total T4 levels were determined in sera as described elsewhere (14, 29). To measure total anti-TSHR antibody titers, 25 µl of sera were combined with 75 µl of human control sera and the antibody concentration was measured using a commercial TRAK kit according to the manufacturer’s instructions (Thermo Scientific B-R-A-H-M-S TRAK human). TSAbs were assessed from 3 µl of serum in stably transfected mouse TSHR Chinese hamster ovary cells and the production of cAMP in the supernatants was quantified by enzyme-linked immunosorbent assay (ELISA) (Enzo, Farmingdale, NY). Total T4, fT4 and fT3 concentrations were determined by ELISA according to the manufacturer’s instructions (DRG Diagnostics GmbH, Marburg, Germany).

### 2.5 Histopathology and immunohistochemistry of thyroids and orbits

Formalin-fixed and paraffin-embedded (FFPE) thyroid glands and orbital tissue were sectioned at 1 µm, deparaffinized, and stained with HE. Investigators blinded to experimental conditions and sample identity performed morphological examination and data collection. Compared to control mouse thyroids, samples were classified as normal, heterogeneous or hyperthyroid. FFPE thyroid sections and orbital tissue were also stained for CD3 (early T-cell marker) using a rabbit polyclonal anti-CD3-IgG antibody (1:25; Dako, #A0452) and positive stained cells were counted with the help of Olympus BX51 microscope.

Orbital tissue sections were also stained with HE as above. ImageJ software was used to quantify areas of nerve, fat and muscle tissue. The percentage of brown adipose tissue (BAT %) in the orbit was determined by measuring the areas of white and brown adipose tissue.

For immunohistochemical staining, paraffin-embedded sections were deparaffinized and subjected to antigen retrieval in citric acid buffer (pH 6.0) or Tris-EDTA buffer (pH 9.0) in a steamer for 45 min, followed by cooling at room temperature for 30 min. They were blocked with pre-blocking solutions for 5 min at room temperature. Sections were incubated overnight at 4 °C with the following primary antibodies: rabbit recombinant monoclonal F4/80 antibody (1:200, Abcam, #111101), rabbit polyclonal anti-TNFα IgG antibody (1:200, elabscience, #E-AB-33121), rat monoclonal anti-CD4 (1:200, eBioscience™, #14-9766-82), rat monoclonal anti-CD8a (1:1000; eBioscience™, #14-0808-80), rabbit polyclonal anti-CCL5 antibody (1:400, Invitrogen, #710001) and visualized using an HRP-conjugated polymer system according to the manufacturer’s instructions (Zytomed Systems). Positive cells were imaged using an Olympus BX51 upright microscope and counted in orbital fat and muscle tissue.

### 2.6 Oil red O staining

To assess the effect of maraviroc (#T6016, Targetmol Chemicals Inc., MA, USA) on adipogenesis, lipid accumulation in human orbital fibroblasts (derived from decompression surgery from patients with TED) was quantified using the Oil Red O (ORO) assay (ref.). Fibroblasts were cultured in standard DMEM with supplements, and adipogenic differentiation was initiated the day after seeding (96 well plate, 1 × 10^4^ cells/well) using DMEM/F-12 (Gibco 11320-033) containing 10% FBS, insulin (10 µg/mL), dexamethasone (1 µM), IBMX (0.5 mM), and L-glutamine. Cells were differentiated for 10 days with medium changes every 2–3 days, while being treated with 2.5 µM maraviroc throughout the differentiation period. After fixation with 4% PFA, cells were stained with ORO working solution, washed, and lipid droplets were imaged by bright-field microscopy. Eluted dye was measured at 500 nm (Agilent BioTek Epoch), and ORO signals from maraviroc-treated cells were expressed relative to untreated controls. Statistical differences were assessed using a paired Student’s t-test.

### 2.7 Immunofluorescence

Sections (1 µm) of paraffin-embedded orbital fat biopsies were deparaffinized and rehydrated. Antigen retrieval was performed by boiling the sections in low-pH citrate buffer (pH 6) for 45 min. Slides were incubated overnight at 4 °C with a mouse anti-CCL5 antibody (1:100, Invitrogen, #710001), followed the next day by incubation with an Alexa Fluor 594 goat anti-rabbit secondary antibody (1:400, Thermo Scientific, #A-11012) for 1 h at room temperature. For double immunofluorescence staining, sections were additionally incubated with antibodies against F4/80 Antibody, anti-mouse, REAfinity (1:50, Miltenyi Biotec, #130-117-509), monoclonal anti-Vimentin (1:500, Sigma, #V5255), or CD3 Antibody, anti-mouse, REAfinity (1:50, Miltenyi Biotec, #130-119-798). Nuclear counterstaining was performed with DAPI (1:1000; Molecular Probes, #D-1306) for 1 min at room temperature.

### 2.8 CCL5 induction and blockade assay

Human Orbital fibroblasts derived from decompression surgery from patients with TED were seeded in 96-well plates (1×10^4^ cells/well) and allowed to adhere overnight. CCL5 production was induced by stimulation with IL-1β. To assess chemokine-receptor blockade, parallel cultures received maraviroc (2,5 µM) together with IL-1ß. After 48h, culture supernatants were collected, and CCL5 levels were quantified using a CCL5 ELISA Kit (Abcam, ab100739) according to the manufacturer’s instructions. Concentrations were calculated from standard curves and compared across treatment groups.

### 2.9 Untargeted lipidomic profiling

Mouse orbital tissue isolated from β Gal, and TSHR plasmid immunized mice treated with maraviroc was shipped to the Metabolomics Innovation Centre (TMIC, Edmonton, AB, Canada) for untargeted lipidomic profiling.

Lipid extraction was performed following a modified Folch liquid-liquid extraction protocol. Briefly, samples were vortexed with NovaMT LipidRep Internal Standard Basic Mix for Tissue/Cells (a mixture of 15 deuterated lipids) and methanol for 20 seconds. Dichloromethane was then added, and the samples were vortexed for an additional 20 seconds. The samples were incubated at room temperature for 10 minutes, followed by centrifugation at 16,000× g for 10 minutes at 4 °C. An aliquot of the resulting organic layer was evaporated to dryness using a nitrogen blowdown evaporator. The dried material was resuspended in 15 µL of mobile phase B (NovaMT MixB, 10 mM ammonium formate in 95% isopropanol: water), vortexed for 1 minute, and then diluted with 135 µL of mobile phase A (NovaMT MixA, 10 mM ammonium formate in methanol: ACN: water 50:40:10 v/v/v). A pooled mixture of all extracts was prepared as quality control samples (QC) and injected into the LC-MS system to monitor instrument performance.

LC-MS analysis was performed in both positive and negative ionization modes using a ThermoFisher Dionex UltiMate 3000 UHPLC (ThermoFisher Scientific) with a C18 Column (Waters Corporation, Milford, MA, USA; 10 cm × 2.1 mm with 1.7 μm particles) coupled with a Bruker Impact II QTOF Mass Spectrometer (Bruker Corporation). Each sample was analyzed in technical duplicate. The chromatograms for the positive and negative ionization injections for each experimental replicate were aligned and combined into a single feature-intensity table using NovaMT LipidScreener (Nova Medical Testing Inc., Edmonton, Alberta, Canada).

A 3-tier identification approach was used to identify the detected peaks, based on MS/MS identification (Tiers 1 and 2) and MS match (Tier 3). Tier 1 included lipids with MS/MS scores ≥ 500, precursor m/z error ≤ 20.0 ppm and 5.0 mDa. Tier 2 consisted of lipids with MS/MS scores < 500, while the other criteria remained the same. Features not identified in Tiers 1 and 2 were searched in the LipidMaps database for putative identification by mass match with an m/z error ≤ 20.0 ppm and 5.0 mDa (Tier 3). Lipid identification followed the guidelines set by the Lipidomics Standards Initiative (31). Further details on lipid classification and nomenclature can be found in other sources (31, 32).

The normalized feature intensities (detected peak intensity/most similar internal standard intensity) were imported into MetaboAnalyst v6.0 (https://www.metaboanalyst.ca) for statistical analysis. Non-informative features (almost constant based on RSD calculation) and those with low repeatability (RSD >30% in QC samples) were removed. The data set was then normalized to the median intensity ratio within each experiment and automatically scaled. For univariate statistics using Volcano plots, lipid species with ≥ ± 1.5 FC for maraviroc treatment/TSHR-immunized groups and raw p-value ≤ 0.05 were considered differentially abundant. In addition to global multivariate analysis, curated metabolite intensity tables were exported from MetaboAnalyst 6.0 and independently filtered to examine class-specific changes in triglycerides (TG) and acylcarnitines (CAR). This approach allowed focused interrogation of lipid classes implicated in adipogenic remodeling within the untargeted dataset.

### 2.10 Statistics

Statistical analyses were performed using Prism 7 (GraphPad Software), including one-way ANOVA for multiple group comparisons and the z-score method. Data are presented as mean ± standard deviation (SD). P-values are indicated as follows: * p ≤ 0.05; ** p ≤ 0.01; *** p ≤ 0.001; **** p ≤ 0.0001. Differences with p > 0.05 were considered not statistically significant and are not shown. To define positivity at the individual-mouse level, we used the upper 99% confidence interval (CI) of the healthy control (β-Gal) group as a threshold. Values above this cutoff are indicated by a dotted line in the figures. The z-score was used to compare results between groups, normalized to the mean of the whole mouse population (reference population) and expressed in standard deviations.

## 3 Results

Maraviroc was administrated to TSHR-immunized mice in order to evaluate the effects of drug in the active state of GD and the associated TED. The experimental design is shown in **Figure 1**.

### 3.1 Treatment does not alter anti-TSHR autoantibody formation in Graves’ disease

**Figure 2:**
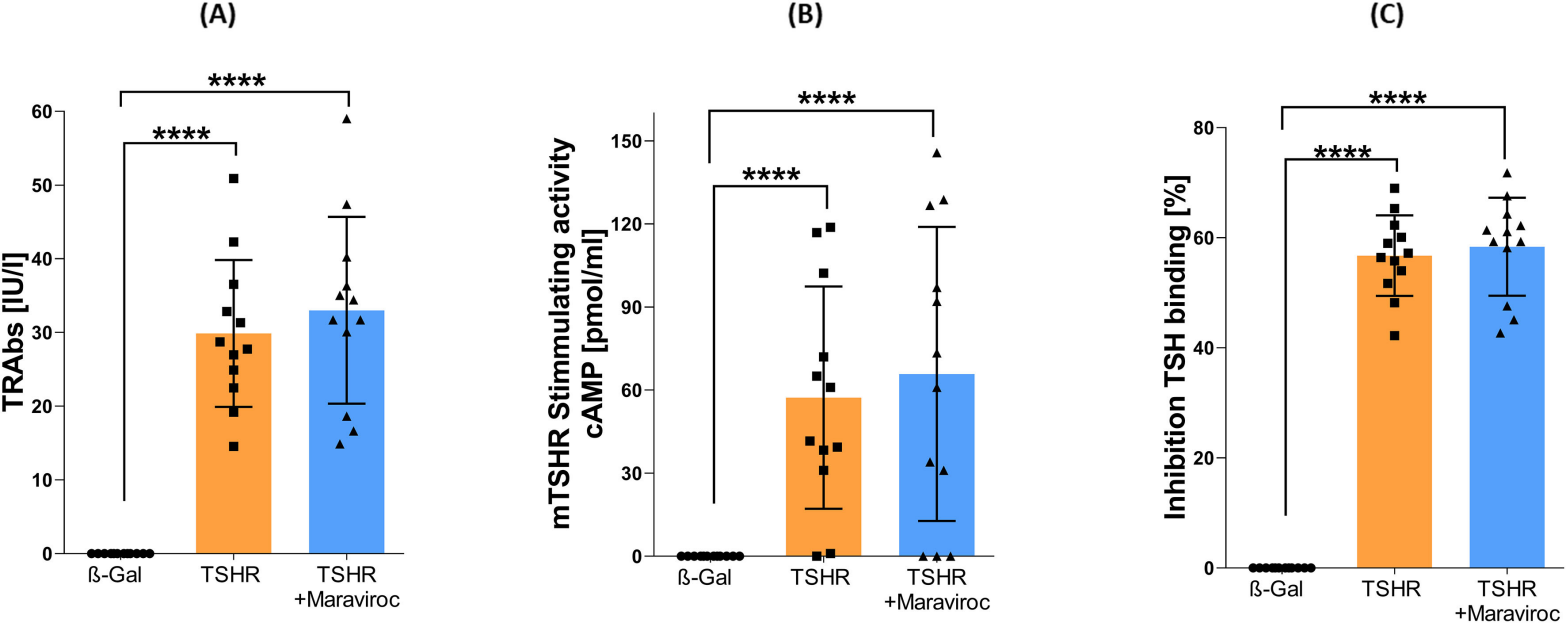
Effect of maraviroc treatment on the formation of autoantibodies against TSHR. **(A)** Total anti-TSHR binding antibody titers (TRAbs) in serum samples from each group (β-Gal, TSHR, and TSHR + maraviroc) were measured. **(B)** The stimulatory activity of anti-mTSHR antibodies (TSAbs) was evaluated by measuring cAMP production in CHO cells treated with the autoantibodies. **(C)** The ability of the autoantibodies to inhibit TSH binding to the TSHR was assessed using an ELISA-based assay. Data are presented as mean ± SD, with n = 12 per group. Statistical significance was determined using one-way ANOVA; *p < 0.05, **p < 0.001, ***p < 0.001, ****p < 0.0001.

To assess the effect of maraviroc on autoantibody formation, total anti TSH receptor antibody (TRAb) level was measured. As expected, healthy ß-Gal-immunized control mice did not produce anti-TSHR antibodies, whereas immunization of mice with the TSHR A-subunit induced high anti-TSHR antibody titers in the sera (**Figure 2A**). In the TSHR-immunized group treated with maraviroc, no statistically significant reduction in median serum TRAb levels from the baseline was observed, indicating that the treatment had no effect on the formation of anti-TSHR autoantibodies (**Figure 2A**). We investigated whether anti-TSHR autoantibodies stimulate cAMP production in target cells and whether maraviroc exerts an influence on this process. The investigations demonstrated that following immunization, eight out of ten mice in the TSHR-immunized mouse group generated antibodies that stimulated the endogenous mouse TSHR (mTSHR) receptor (**Figure 2B**). Maraviroc did not change the activation of endogenous TSHR and the formation of cAMP (**Figure 2B**). Subsequently, the antibodies induced by immunization were also observed to block TSH binding, underscoring the functional significance of these antibodies (**Figure 2C**).

### 3.2 Maraviroc does not reduce autoimmune hyperthyroidism in the early state of the disease

**Figure 3:**
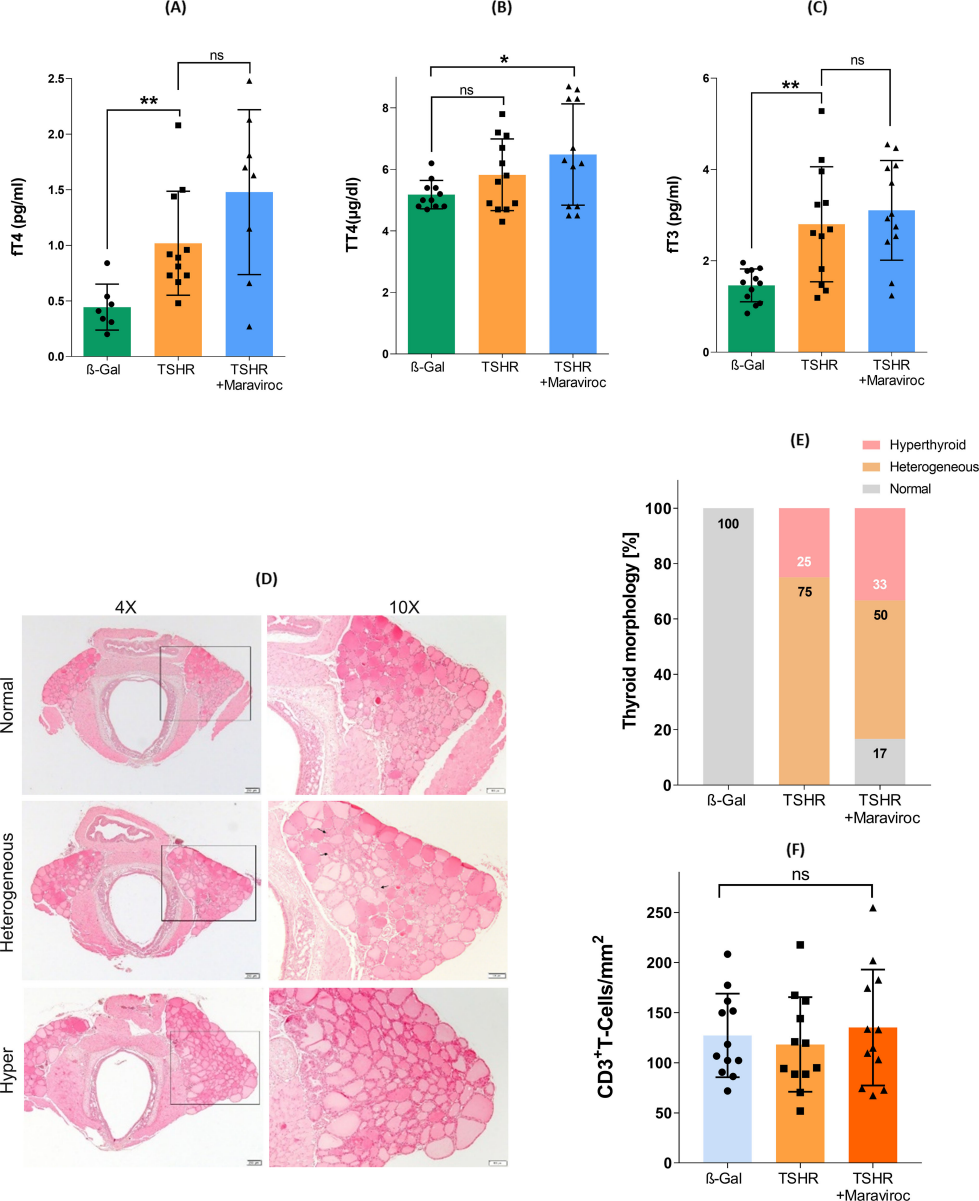
Assessment of thyroid function and morphology following maraviroc treatment. **(A–C)** Serum levels of free and total T4 as well as free T3 were measured using ELISA. **(D)** Thyroid glands from all mice in each group were fixed, paraffin-embedded, and sectioned (1 µm) through the mid-region of the gland, followed by H&E staining. Representative images display normal, heterogeneous, or hyperplastic thyroid morphology at 4× and 10× magnification. Arrows indicate regions of hyperplasia within otherwise normal thyroid architecture. **(E)** Thyroid morphology was categorized as normal, heterogeneous, or hyperplastic, and the percentage distribution of each type was calculated per group. **(F)** Mid-thyroid sections were immunohistochemically stained for CD3, and CD3^+^ T cells were quantified. Results are shown as mean ± SD for **(A–C, F)**, and as representative images in **(D)**, with n = 12 per group. Statistical significance was determined using one-way ANOVA; p < 0.05 (*), p < 0.001 (**).

We assessed the effect of maraviroc on the degree of hyperthyroidism by measuring serum thyroid hormone levels, fT3, fT4 and TT4 and by evaluating thyroid histopathology (**Figure 3**). The fT4 and TT4 levels of the TSHR immunized groups did not significantly differ from those of the healthy control ß-Gal immunized group (**Figures 3A, B**). In contrast, the fT3 concentration was significantly increased in the TSHR immunized group compared to the healthy control ß-Gal immunized group (**Figure 3C**). The administration of maraviroc did not prevent the elevation in the serum concentrations of fT3 in TSHR immunized mice (**Figure 3C**) compared to control ß-Gal immunized group. Maraviroc did not alter the serum concentrations of fT4 and TT4. No significant alterations in thyroid hormone levels were observed in TSHR-immunized compared to TSHR-immunized mice following maraviroc treatment.

To identify morphological changes indicative of autoimmune hyperthyroidism, thyroid gland tissue sections were stained with H&E. **Figure 3D** shows three morphological categories of thyroid. The gland presents round or oval follicles when it is in a normal physiological state. The core of the gland contains the smaller follicles, while the peripheral zone contains the larger follicles. The walls of the follicle are lined with a single layer of cuboidal thyroid epithelium (**Figure 3D**). The thyroid undergoes morphological changes in a hyperthyroid state, including asymmetric thyroid lobes, resizing of the follicles, the appearance of irregularly shaped follicles, multilayer epithelium, and changing the amount of colloid that fills the follicles. Some of the glands displayed a heterogeneous morphology with a mixture of areas of normal appearance and areas of hyperplastic follicles (**Figure 3D**).

In the TSHR-immunized group, 25% of mice exhibited hyperthyroid morphology, while the remaining 75% displayed heterogeneous thyroid architecture, indicating that 100% of mice showed some degree of thyroid pathology. Treatment with maraviroc reduced this overall pathological incidence to 83%, with 17% of mice displaying a normal thyroid morphology (**Figure 3E**). These findings suggest that maraviroc may modestly mitigate thyroid gland morphological alterations associated with hyperthyroidism, although its impact on systemic thyroid function remains limited.

CD3 staining of thyroid did not reveal T‐cell infiltration into the interfollicular connective tissue in TSHR‐immunized mice. No significant differences were observed between the TSHR-immunized and maraviroc-treated groups compared to the healthy ß-Gal-immunized control group (**Figure 3F**).

### 3.3 Treatment does not alter the body weight

**Figure 4:**
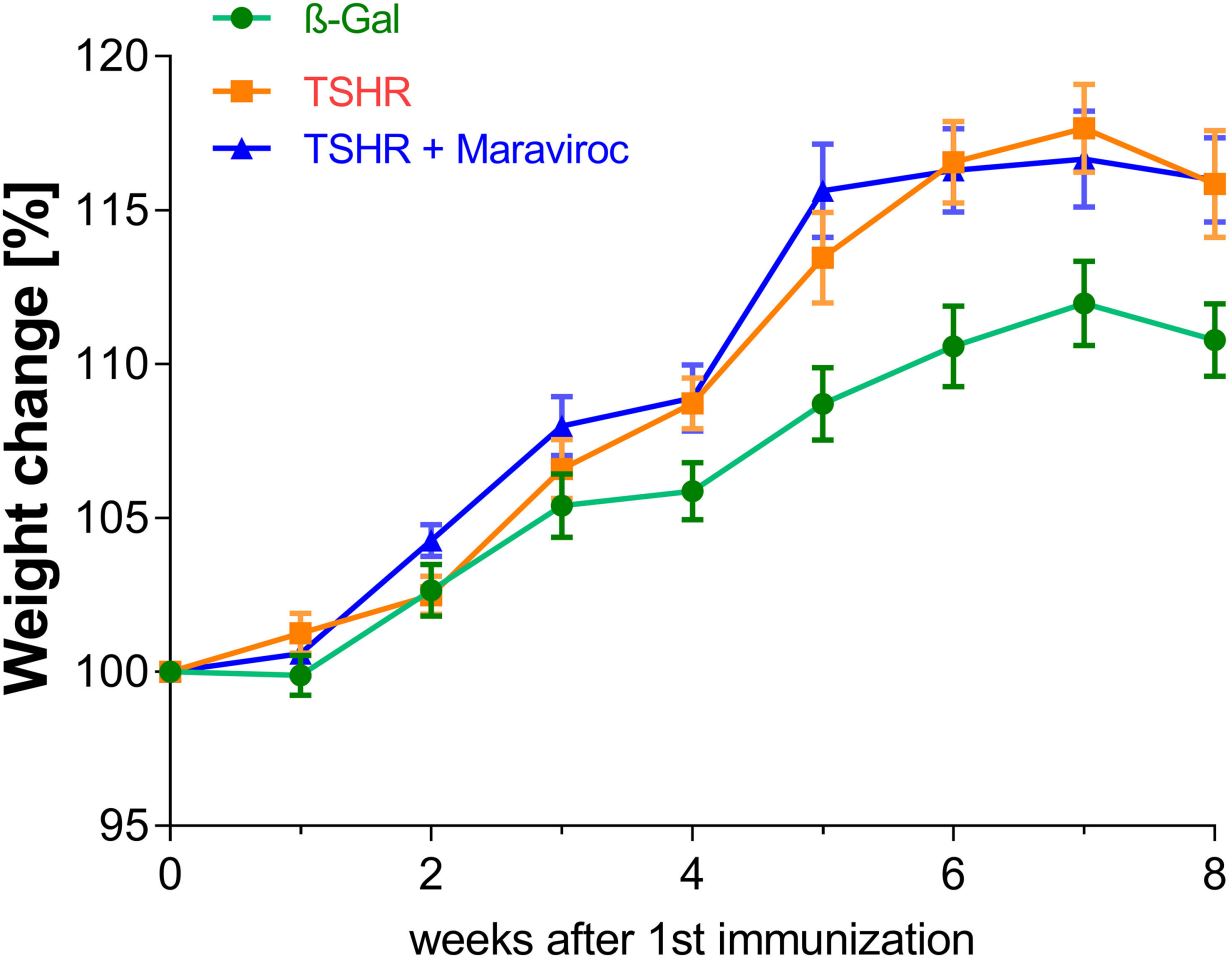
Physiological *in vivo* parameters in response to maraviroc treatment. Body weight of all mice was monitored daily throughout the study period. Statistical analysis was performed by calculating the area under the curve (AUC), followed by Student’s t-test. n = 12 per group.

A reduction in body weight may serve as a physiological indicator of stress in animals or because of adverse effects associated with pharmacological agents, including maraviroc. Body weight of all mice was measured daily during the study as maraviroc may alter energy metabolism. As expected from previous TSHR-immunization experiments in our laboratory, TSHR-immunized mice showed an overall increase in body weight compared with β-Gal control animals. This weight gain corresponded with an increase in daily food intake typically observed in hyperthyroid mice (33). Treatment with maraviroc did not lead to a reduction in body weight in the TSH-immunized group compared to the untreated ß-Gal control mice (**Figure 4**).

### 3.4 Maraviroc improves TED

**Figure 5:**
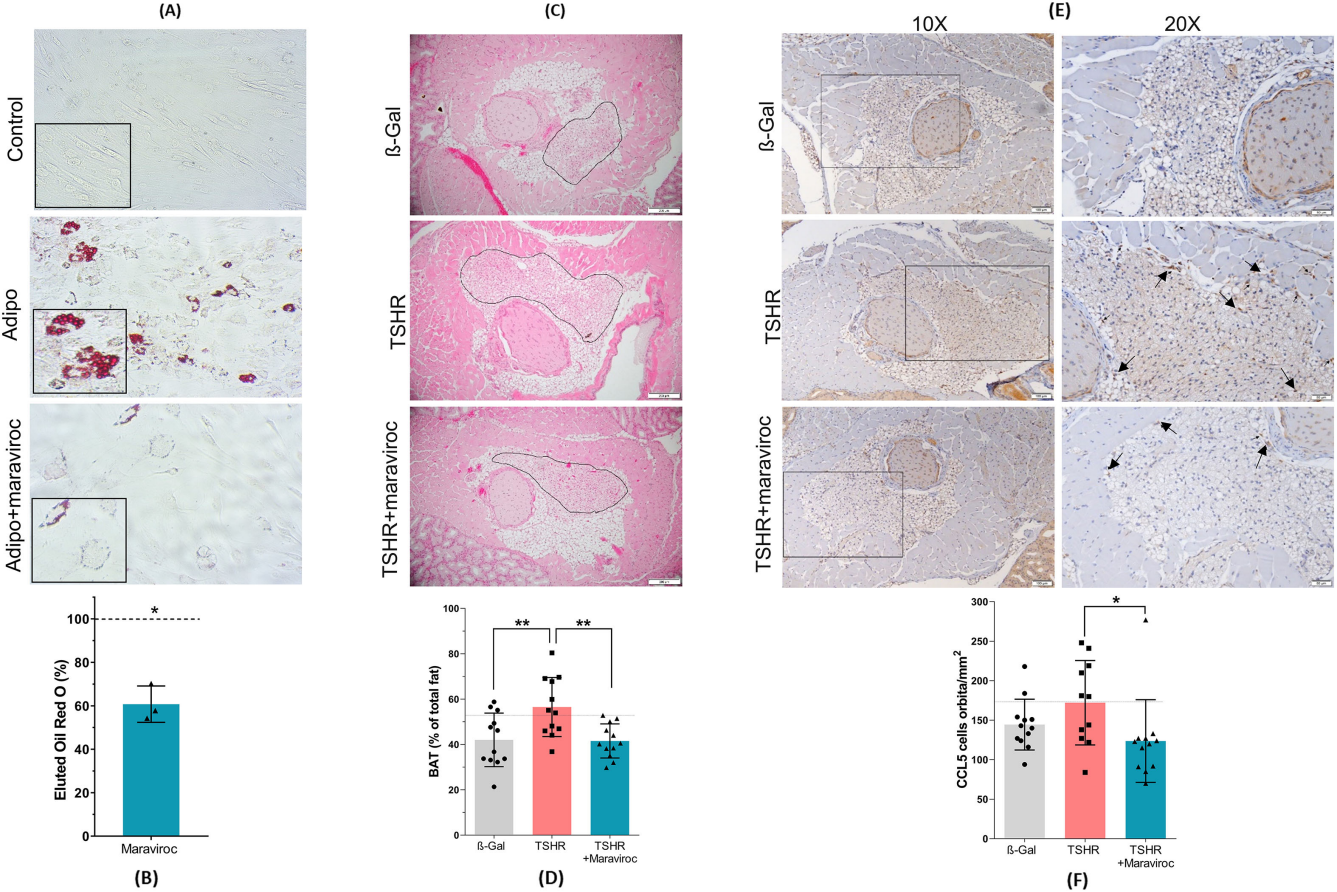
Effect of maraviroc on orbital fibroblasts *in vitro* and in mice with experimental Graves’ disease and thyroid eye disease. **(A–F)** Orbital fibroblasts were isolated from orbital tissue samples and used for *in vitro* adipogenesis assays. In addition, whole orbital tissues were fixed, paraffin-embedded, and consecutive mid-orbital sections were stained with H&E and anti-CCL5 antibodies. **(A)** Representative images of Lipid droplet formation are shown (20x magnification) after fixation and Oil Red o Staining (ORO). **(B)** ORO was eluted to calculate the accumulation of lipids into the cells relative to control. Statistical significance used paired Student’s t test. **(C)** Representative images of brown adipose tissue are shown (20× magnification). The brown adipose tissue is indicated by a polygonal outline in the images. **(D)** The area of brown adipose tissue was quantified and expressed as a percentage of total orbital fat. **(E)** Representative images of orbital sections stained for CCL5 are shown at 10× and 20× magnification, with arrows indicating CCL5^+^ cells **(F)** The number of CCL5^+^ cells was quantified across the entire orbital section. Results are presented as mean ± SD for **(B, D, F)**, and as representative images for **(A, C, E)**. n= 12 animals/group: β-Gal, TSHR, and TSHR + maraviroc. Statistical significance was determined using one-way ANOVA; p <0.05 (*), p < 0.001 (**).

**Figure 6:**
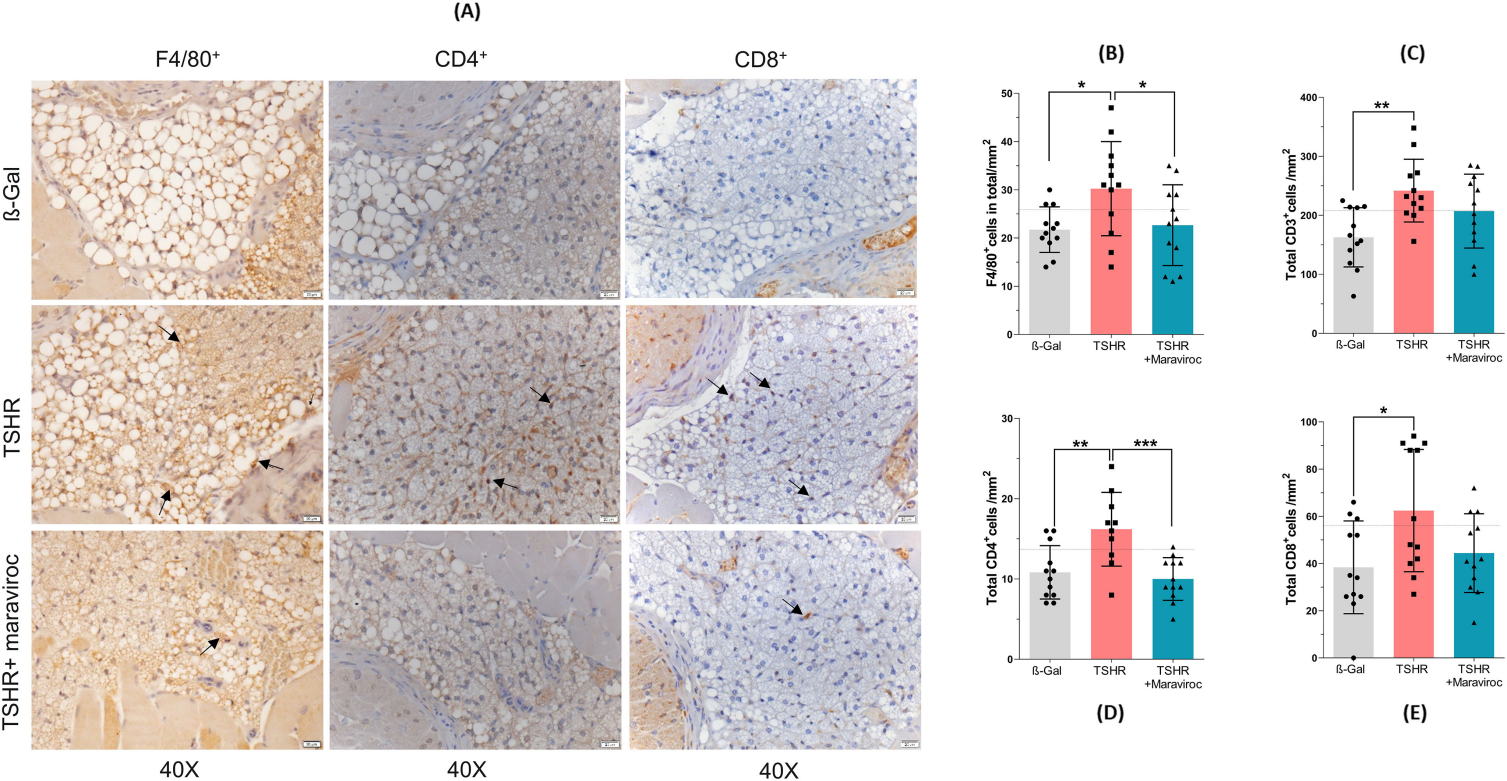
Effect of maraviroc on orbital immune cell infiltration. **(A)** Orbital tissues were collected, fixed, paraffin-embedded, and serial mid-orbital sections were stained with antibodies against F4/80, CD3, CD4, and CD8. Representative images of F4/80, CD4, and CD8 immunostaining (40× magnification) are shown. Arrows indicate F4/80^+^ macrophages and CD4^+^ or CD8^+^ T cells within the orbital tissue. **(B–E)** Quantification of immune cell infiltration across the entire orbital section: **(B)** F4/80^+^ macrophages, **(C)** CD3^+^ T cells, **(D)** CD4^+^ T cells, and **(E)** CD8^+^ T cells. Data are presented as mean ± SD (n = 12 per group: β-Gal, TSHR, and TSHR + maraviroc). Statistical significance was assessed using one-way ANOVA; p < 0.05 (*), p < 0.001 (**).

To assess whether and to what extent maraviroc reduces orbitopathy, serial sections of orbits were stained for CD3^+^, CD4^+^ and CD8^+^T cells, tissue macrophages, brown adipose tissue and TNFα (**Figure 5**, **6**; **Supplementary Figure 3**).

To determine whether maraviroc affects adipogenic differentiation in OFs, cells were cultured for 10 days in adipogenic medium. As expected, differentiated OFs developed prominent lipid vacuoles, whereas untreated controls showed minimal adipogenic activity and virtually no lipid droplet formation. OFs treated with maraviroc during differentiation exhibited markedly fewer and substantially smaller lipid droplets (**Figures 5A**; **Supplementary Figure 2A**). Quantification of Oil Red O staining revealed that maraviroc reduced lipid accumulation by approximately 40% compared with fully differentiated OFs (**Figures 5B**; **Supplementary Figure 2B**). *In vivo*, the orbital tissue of the immunized mice exhibited a higher proportion of brown adipose tissue (**Figures 5C, D**). The administration of maraviroc resulted in the normalization of the quantity of brown adipose tissue observed in the TSHR-immunized group, which subsequently exhibited no discernible difference from the healthy ß-Gal group (**Figure 5D**). A representative example of a hematoxylin and eosin staining of the orbital tissue for brown adipose tissue is presented in **Figure 5C**. CCR5-CCL5 axis plays a crucial role in the recruitment and activation of immune cells during inflammatory responses (34, 35). However, function of CCR5/CCL5 in TED is presently unknown. In this regard, orbital tissue sections from these groups (ß-Gal, TSHR and TSHR treated with maraviroc) were stained for CCL5 and examined for the presence and distribution of CCL5-positive cells (**Figures 5E, F**). Representative images in **Figure 5E** reveal that expression of CCL5 is notably higher in the TSHR group compared to the ß-Gal control group. To quantify the expression levels of CCL5, we further assessed the density of CCL5-positive cells in the tissue sections. As shown in **Figures 5E**, the TSHR group exhibited a significantly greater number of CCL5-positive cells compared to the ß-Gal control group, with an average of 200 CCL5-positive cells per mm². The TSHR group treated with maraviroc showed a marked reduction in the number of CCL5-positive cells (**Figure 5E**). To identify the cellular sources of CCL5 in orbital tissue, we performed double-immunofluorescence staining using CCL5 in combination with markers for fibroblasts (vimentin), macrophages (F4/80), and T cells (CD3) (**Supplementary Figure 1A**). In all three staining panels, we detected double-positive cells, indicating that fibroblasts, macrophages, and CD3^+^ T cells contribute to CCL5 expression within the orbit. To further evaluate CCL5 production in orbital fibroblasts, we performed a CCL5 ELISA on cultured OF stimulated with IL-1β (**Supplementary Figure 1B**). IL-1β stimulation resulted in a marked increase in CCL5 secretion, while maraviroc treatment reduced CCL5 levels back to baseline. These results confirm that orbital fibroblasts are a relevant source of CCL5 and that CCL5 secretion is sensitive to maraviroc treatment.

Macrophage infiltration was assessed by immunostaining with F4/80 (**Figures 6A, B**) and TNFα **Supplementary Figure 1D**). F4/80 is a cell surface molecule that is highly expressed on murine monocytes and tissue macrophages (20), while TNFα is a pro-inflammatory cytokine known to be highly produced by macrophages during inflammation, injury or infection, and thus mainly indicative of pro-inflammatory M1 macrophages (36).

Immunohistochemical analysis of F4/80 (**Figure 6A**) and TNFα (**Supplementary Figure 3**) revealed a marked increase in TNFα-positive cells and macrophage infiltration within the orbital tissue of the TSHR-immunized group. While maraviroc treatment did not significantly alter the number of TNFα-positive cells, it led to a significant reduction in F4/80^+^ macrophage infiltration in TSHR-immunized mice (**Figures 6A, B**). One possible explanation is that maraviroc, as a CCR5 antagonist, selectively inhibits CCR5-mediated macrophage recruitment without affecting TNFα expression, which may be regulated through CCR5-independent pathways.

Immunostaining for CD3^+^ T-cells revealed a marked infiltration of T-cells into the orbital fat and muscles in TSHR-immunized mice, in comparison to those immunized with ß-Gal (**Figure 6A, C**). Maraviroc demonstrated some improvement in T-cell infiltration in the TSHR mice, although this was not statistically significant due to the relatively high degree of variability. To further characterize the T-cell response, CD4^+^ and CD8^+^ T-cell subsets in the orbital tissue were examined by immunohistochemistry (**Figures 6A, D, E**). In TSHR-immunized mice, CD4^+^ T cells were increased in the orbital tissue compared with controls, and maraviroc treatment reduced CD4^+^ T-cell levels to control values (**Figure 6D**). CD8^+^ T cells were likewise elevated in the TSHR model and showed a tendency toward reduction following maraviroc treatment. Total CD3^+^ T-cell numbers were higher in immunized mice and were not markedly altered by maraviroc administration.

### 3.5 Maraviroc improves orbital remodeling and the outcome of TED

In order to compare the disease outcomes between the different mouse groups, we used a Z-score method, as described earlier (14, 22, 29).

**Figure 7:**
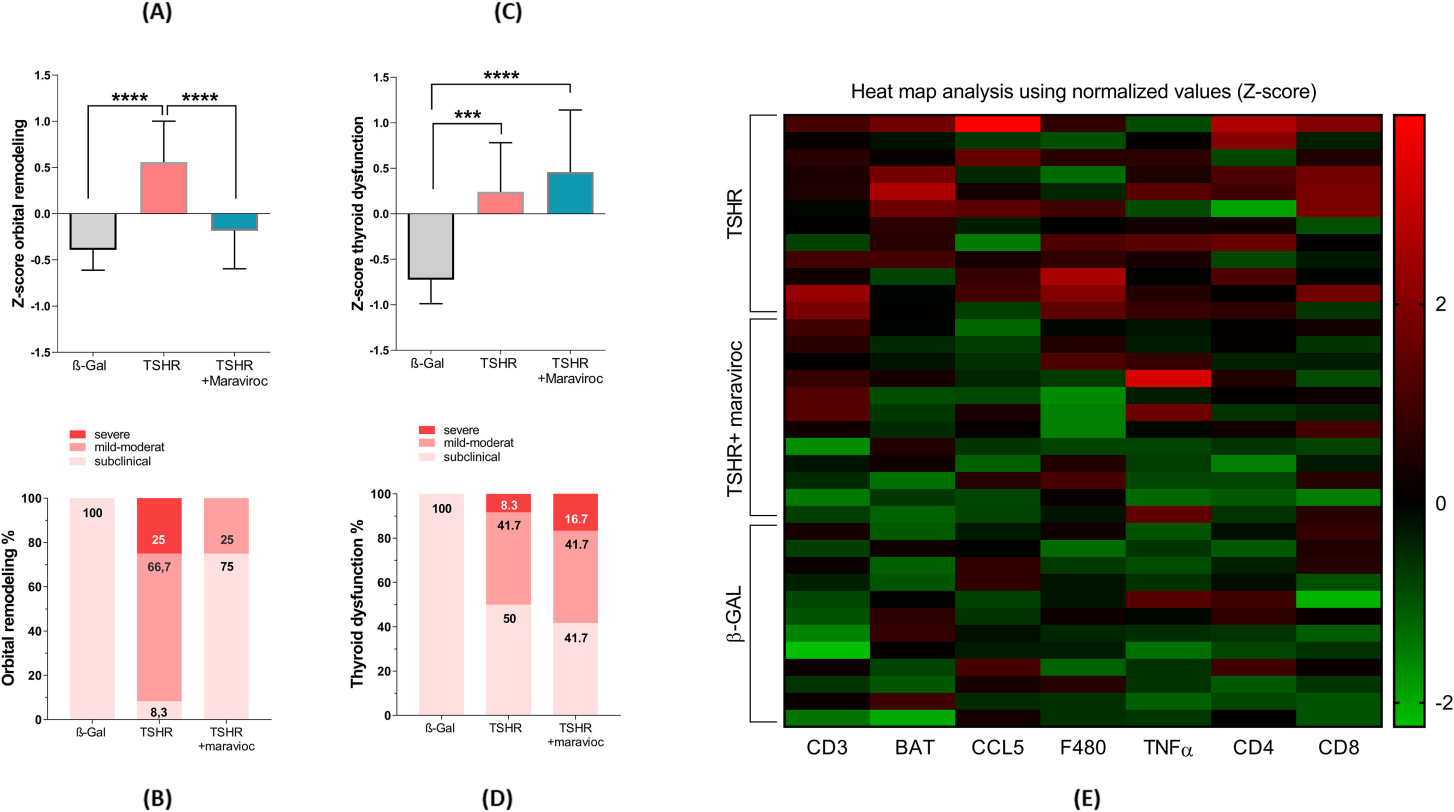
Total disease outcome of untreated and maraviroc-treated immunized mice. (**A–E)** Results from various experiments were compiled, normalized, and analyzed using the Z-score method. The Z-score represents the number of standard deviations from the mean of the entire mouse cohort and is expressed in arbitrary units. **(A)** The Z-score for thyroid dysfunction was calculated based on the following parameters: stimulatory activity of anti-TSHR autoantibodies and total anti-TSHR binding antibody titers (**Figure 2**), serum T4 levels and thyroidal CD3^+^ T-cell infiltration (**Figure 3**), and body weight change (**Figure 4**). **(B)** Disease classification was determined using Z-score values. Mice were categorized as follows: Subclinical disease (Z-score < 0): Mice that did not exhibit significant autoimmune hyperthyroidism or TED, though they may have developed TSHR antibodies. Clinical disease (Z-score > 0): Mice exhibiting clinical disease during the study, further classified as mild to moderate (0 < Z < 1) or severe (Z > 1). The number of mice in each category is expressed as a percentage. **(C)** The Z-score for orbital remodeling was calculated from measurements of orbital CD3^+^ T-cell infiltration (**Figure 6**), brown adipose tissue expansion (**Figure 5**), orbital macrophage infiltration (**Figure 6**), and CCL5 levels (**Figure 5**). **(D)** Disease classification for orbital remodeling followed the same Z-score thresholds as in **(B)**. **(E)** A heat map analysis of normalized values is shown to represent overall disease outcome across all parameters. Data are presented as mean ± SD for panels **(A, C)**, and as percentage-based disease classifications in **(B, D)**. n = 12 per group. Statistical significance was assessed by one-way ANOVA; p < 0.05 (*), p < 0.001 (**), p < 0.001 (***), p < 0.0001 (****).

To comprehensively assess disease severity, we combined normalized and summarized multiple histological and immunological parameters to generate composite Z-scores reflecting either thyroid dysfunction or orbital tissue remodeling on a per-mouse basis. For evaluation of orbital pathology, four key parameters were included in the Z-score calculation: CD3^+^ T cell infiltration (**Figure 6**), brown adipose tissue expansion (**Figure 5**), orbital macrophage (F4/80^+^) infiltration (**Figure 6**), and CCL5 expression (**Figure 5**). As expected, TSHR-immunized mice developed significant orbital pathology compared to healthy β-Gal-immunized controls (**Figure 7A**). Maraviroc treatment significantly ameliorated orbital tissue remodeling in the TSHR-immunized group. Notably, the maraviroc-treated mice did not statistically differ from β-Gal controls, indicating a normalization of orbital tissue architecture and immune infiltration (**Figure 7A**). These findings underscore the therapeutic potential of maraviroc in attenuating key pathological features of TED. Furthermore, the individual Z-scores for each mouse were categorized into different severity levels, similar to the disease classification used for assessing TED severity in patients (5). Z-scores indicate the following: Z-score 0 indicates healthy/subclinical orbital manifestation; Z-score 1 one indicates a mild-moderate disease and a Z-score >1 represents a severe disease. Clinical classification revealed that two thirds of the TSHR-immunized group (66.7%) developed mild-to-moderate TED, while 25% developed severe TED symptoms (**Figure 7B**). Maraviroc significantly improved this orbital remodeling and 75.0% of the mice were scored healthy, and only 25.0% mice were scored with mild-moderate TED (**Figure 7B**).

To assess thyroid dysfunction, five data sets were incorporated into the Z-score: the stimulatory activity of anti-TSHR autoantibodies (**Figure 2**), total anti-TSHR binding antibody titers (**Figure 2**), and T4 levels (**Figure 3**), thyroidal CD3^+^ T-cell infiltration (**Figure 3**), and changes in body weight (**Figure 4**). As shown in **Figures 7C, the** TSHR-immunized group exhibited pronounced thyroid dysfunction compared to the healthy ß-Gal control group (**Figure 7C**). In the TSHR-immunized group, 50% of the mice developed autoimmune hyperthyroidism, with 41.7% exhibiting mild to moderate thyroid dysfunction and 8.3% presenting with severe thyroid dysfunction (**Figure 7D**). In contrast, 100% of the control group maintained normal or subclinical thyroid function (**Figure 7D**). Treatment with maraviroc in the TSHR-immunized mice had no effect the thyroid morphology. To visualize these alterations, we generated heat maps based on the Z-score values (**Figure 7E**). The heat map clearly revealed a marked difference between the TSHR non-treated group and the TSHR group treated with maraviroc. In the TSHR non-treated group, elevated Z-scores for brown adipose percentage, inflammatory markers and immune cell markers, such as CCL5, indicated a heightened inflammatory state associated with the disease. In contrast, the TSHR group treated with maraviroc exhibited significantly reduced Z-scores for these markers, suggesting that maraviroc treatment led to a dampened immune response and improved control over inflammation.

### 3.6 Treatment changes lipid profiling pattern

**Figure 8:**
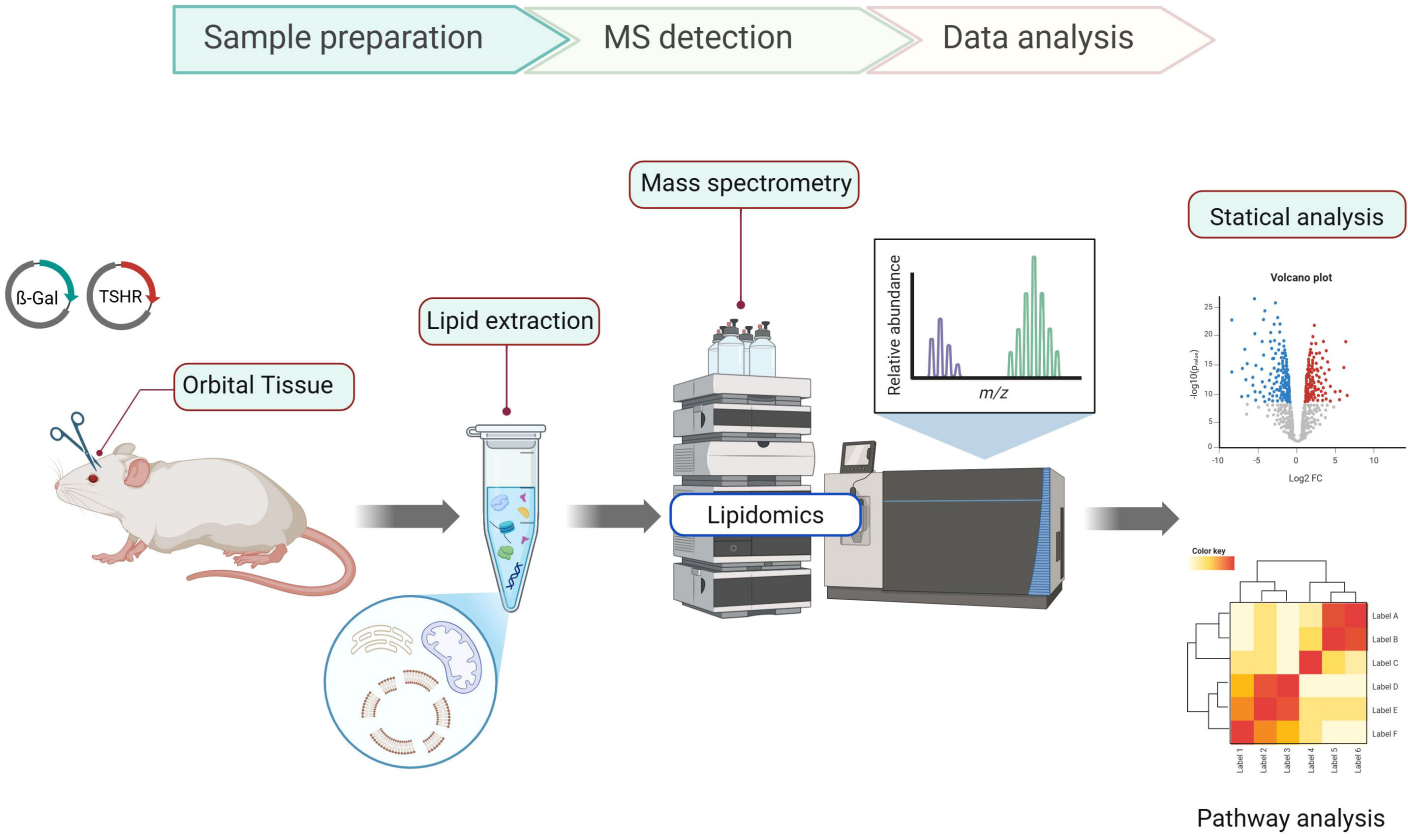
Untargeted lipidomics workflow from orbital tissue extraction to mass spectrometric and statistical analysis.

To generate a more complete picture of the orbital tissue lipid profile in TSHR and TSHR immunized treated with maraviroc, ultra-high performance liquid chromatography (UHPLC) coupled with a quadrupole time-of-flight (QTOF) mass spectrometer were used for the untargeted lipidomics measurements. The combination of these two technologies enables highly efficient separation and precise identification of analytes. UHPLC with a C18 column separates compounds based on the polarity of the lipid headgroup and the hydrophobicity of the fatty acid carbon chains (including their length, saturation, and unsaturation levels). The role of the QTOF is to identify and quantify the compounds based on their mass-to-charge ratios (m/z) and fragmentation patterns. **Figure 8** depicts the lipidomics workflow from the sample extraction, to the mass spectrometric and statistical analysis. Using the 3-tier lipid identification method, based on the lipid MS/MS match scores and the precursor m/z errors, a total of 827 lipids were identified with high confidence in tier 1, 114 lipids were identified with high confidence in tier 2, and 3919 features were putatively identified in Tier 3 at the species level with m/z error threshold of 20.0 ppm and 5.0 mDa.

**Figure 9:**
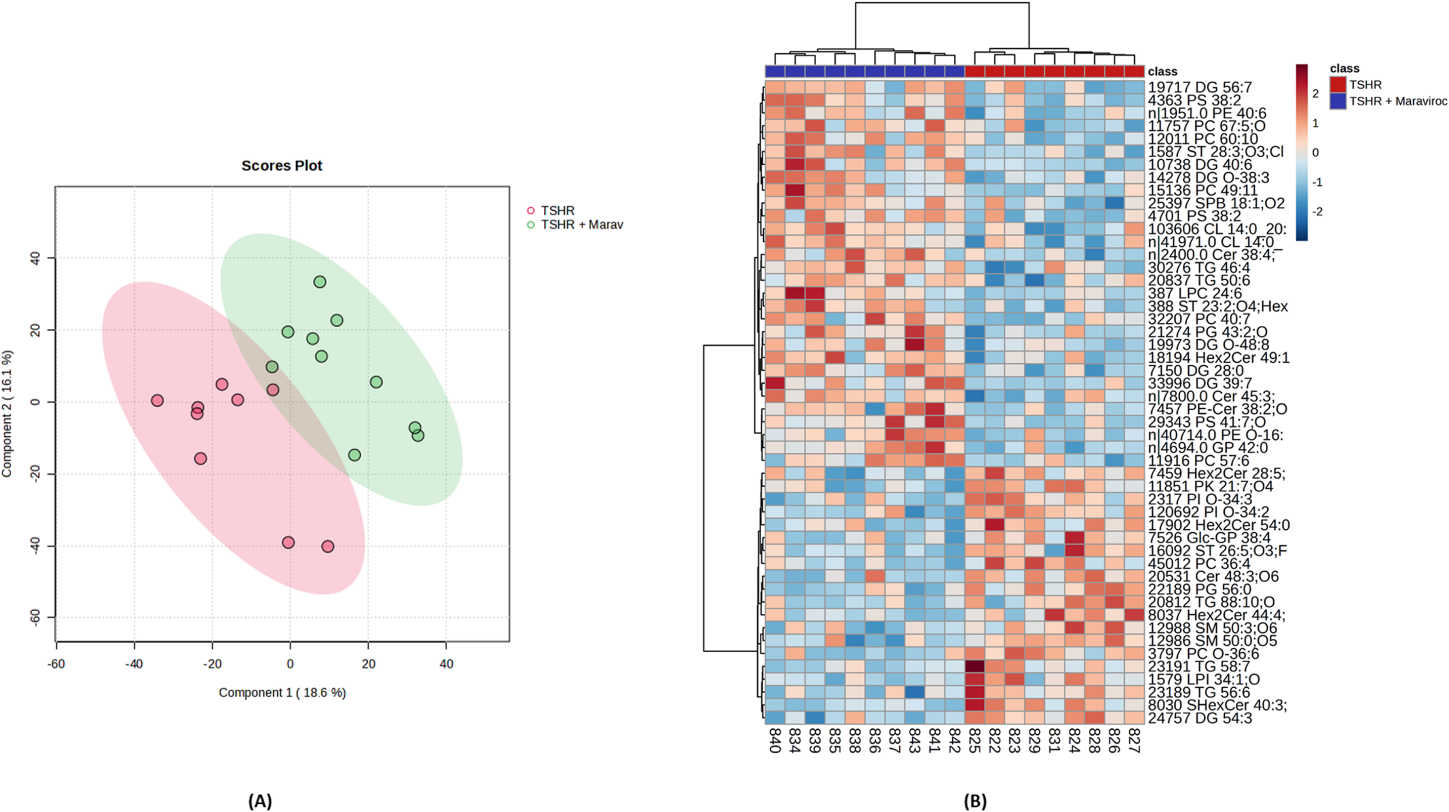
Lipidomics analysis of orbital tissues from TSHR-immunized mice with and without maraviroc treatment. **(A)** Partial Least Squares Discriminant Analysis (PLS-DA) showing the separation of lipid profiles between TSHR-immunized mice and TSHR-immunized mice treated with maraviroc. **(B)** Heatmap of the top 50 differentially expressed lipids. Red indicates upregulation, and blue indicates downregulation. Each lipid is annotated with its corresponding subclass.

**Figure 9A** shows the partial least-squares discriminant analysis (PLS-DA) 2D score plot for the untargeted lipidomics of the UPLC-QTOF separation. The degree of separation in the lipidome profiles between the TSHR and maraviroc-treated groups is visualized, with a clear distinction between the two groups. This indicates that maraviroc has a significant impact on lipid composition. This finding is further reflected in the heatmap (showing the top 50 affected lipids) presented in **Figure 9B**. Blue indicates downregulated lipids, while red indicates upregulated lipids. What is evident from the heatmap is that both groups exhibit distinct lipid expression patterns, highlighting that immunization following maraviroc treatment has caused significant changes in lipid metabolism.

**Figure 10:**
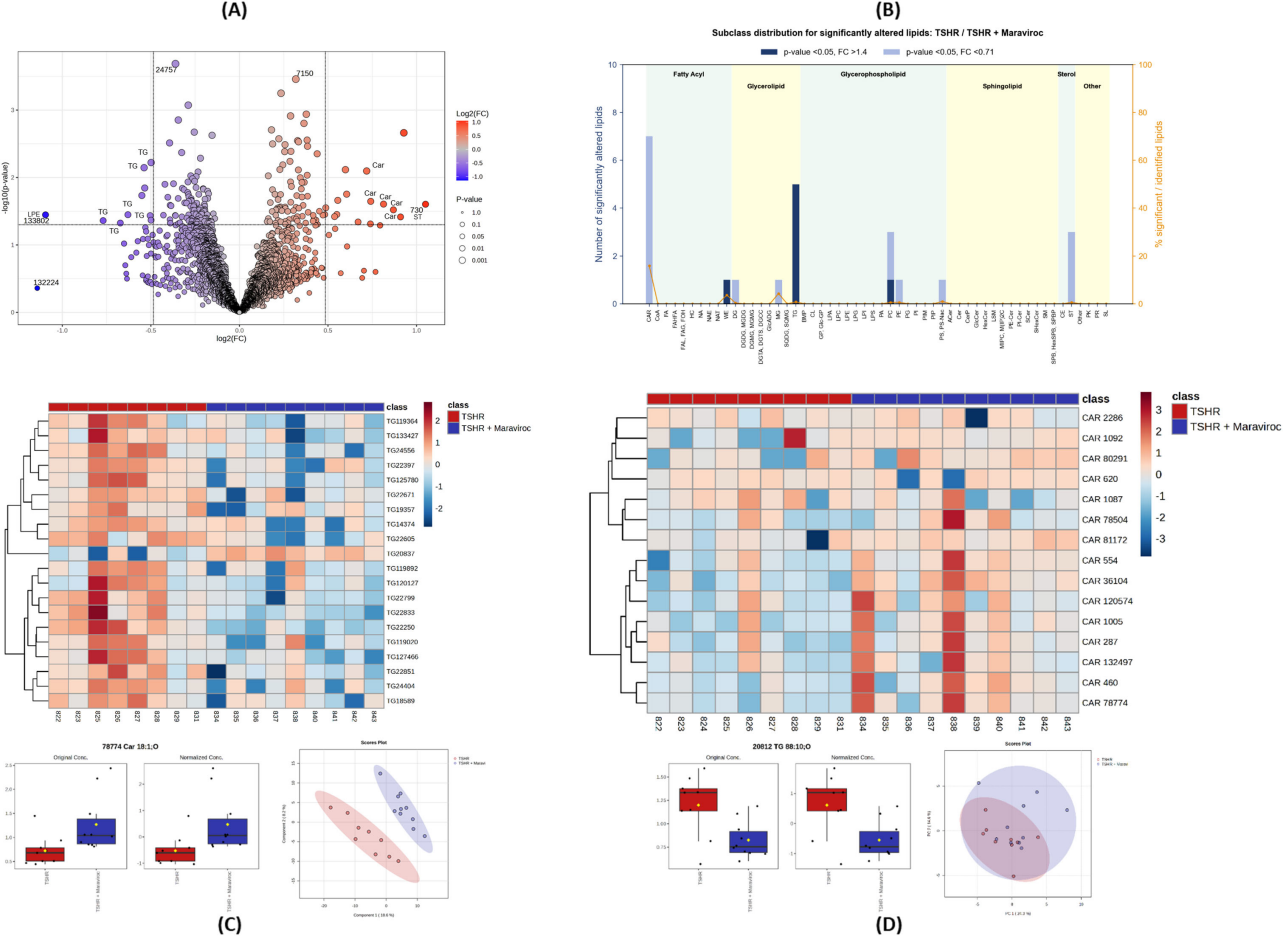
Differential lipid expression between maraviroc-treated and untreated TSHR-immunized mice. **(A)** Volcano plot showing significantly altered lipids between TSHR-immunized mice and those treated with maraviroc. A fold change (FC) thresh-old of 1.4 and a p-value threshold of 0.05 were applied. Red dots indicate lipids upregulated in the maraviroc-treated group, while blue dots indicate downregulated lipids; names of all significantly altered species are shown on the plot. **(B)** Relative Distribution of Significantly Altered Lipid Classes. Bar graph illustrating the relative distribution of lipid subclasses that were significantly altered between groups. The lipid classes are abbreviated as follows: Carnitine/Acylcarnitines (CAR), Ceramides (Cer), Di-acylglycerols (DG), Monoacylglycerols (MG), Phosphatidylcholines (PC), Phosphatidylethanolamines (PE), Phosphatidylserines (PS), Triacylglycerophosphoserines (PS-NAc), Sterols (ST), Triacylglycerols (TG), Wax Esters and Diesters (WE). **(C, D)** Heatmap of selected TG and CAR species derived from the untargeted lipidomics dataset. Values represent z-score-normalized metabolite intensities, with rows corresponding to individual lipid species and columns to individual samples. Hierarchical clustering was applied to both lipid species and samples, which are annotated by treatment group (TSHR and TSHR + maraviroc). Representative histograms illustrate a TG species that is reduced and a CAR species that is increased following maraviroc treatment. Principal component analysis (PCA) was performed on curated metabolite intensity tables focusing on TG and CAR species.

Based on volcano plot analysis of the UPLC–QTOF untargeted lipidomics data, more than 20 lipid species were differentially expressed between TSHR control and maraviroc-treated retro-orbital tissues (**Figure 10A**). Applying a threshold of fold change (FC) > 1.4 and p ≤ 0.05, a total of 25 significantly altered lipids were identified. These differentially regulated metabolites spanned multiple lipid classes, with prominent representation of triglycerides (TG), acylcarnitines (CAR), sterols (ST), and phosphatidylcholines (PC), as summarized in the class distribution bar chart (**Figure 10B**). Inspection of the volcano plots revealed a clear directional pattern: TG species were predominantly decreased in maraviroc-treated samples and clustered on the left side of the plot, whereas CAR species were among the most abundant lipids increased following maraviroc treatment and localized to the opposite side of the plot. Box plot analyses of selected significantly altered lipids illustrate internal standard-normalized intensity differences between treatment groups (**Figures 10C, D**). To further assess whether these changes reflected coordinated lipid-class remodeling rather than effects driven by individual metabolites, we performed an independent, targeted-like re-analysis of the untargeted dataset focusing specifically on biologically relevant lipid classes implicated in adipogenic metabolism, namely TG and CAR. Curated and filtered metabolite intensity tables were interrogated at the class level. Heatmap visualization demonstrated a coordinated reduction across multiple TG species accompanied by a concomitant increase in multiple CAR species in maraviroc-treated tissues. Consistently, principal component analysis (PCA) based on TG and CAR species revealed treatment-associated clustering trends, indicating that these lipid classes contribute substantially to the observed separation between groups (**Figure 10C, D**). Together, these analyses indicate that maraviroc treatment is associated with coordinated, class-level lipid remodeling characterized by reduced triglyceride abundance and increased acylcarnitine levels, rather than isolated changes in individual lipid species.

## 4 Discussion

In this study, we investigated the effects of maraviroc, an FDA-approved CCR5 receptor antagonist approved for antiretroviral medication for treatment of human immunodeficiency virus (HIV) (36, 37), on a mouse model of GD and the associated condition TED. Our findings suggest that maraviroc has a notable therapeutic effect, particularly in the context of orbital remodeling and inflammatory processes associated with GD, though its impact on autoimmune hyperthyroidism is modest. Furthermore, we showed that treatment with maraviroc did not alter body weight in the TSH-immunized mice compared to control mice (**Figure 4A**). This suggests that maraviroc treatment does not induce significant metabolic stress or adverse effects related to energy balance in this model. This finding is crucial for interpreting the results of therapeutic trials, as weight loss can be a confounding factor in assessing drug efficacy and toxicity. The absence of weight change also indicates that maraviroc’s mechanism of action does not significantly affect overall metabolic homeostasis, which is an important consideration when evaluating its long-term safety and therapeutic potential.

### 4.1 Reproducibility and reliability of the mouse model

Subsequent studies have demonstrated that the mouse model employed in the present study is highly reproducible across varying environmental conditions and consistently reflects key pathological features of TED that are comparable to the clinical manifestations observed in patients (22, 26). Our model followed the same treatment timeline as the early intervention group in the linisitinib trial (14). The orbital remodeling in our TSHR-immunized mice was comparable to that reported by Gulbins et al., with a similar ratio of mildly/moderately to severely affected animals.

### 4.2 Maraviroc and autoimmune hyperthyroidism

In this study, we assessed the effects of maraviroc on hyperthyroidism by measuring serum thyroid hormone levels and evaluating thyroid histopathology. Maraviroc application did not affect thyroid function and morphology. Changes in thyroid morphology are mainly mediated by the direct action of stimulating TSHR autoantibodies. As demonstrated by the TRAb levels measured in this study, antibody production also remained largely unaffected by CCR5 blockade with maraviroc. This is consistent with the fact that B cells, the primary antibody-producing cells, express little to no CCR5 (38). CCR5 is one of two main co-receptors on CD4^+^ T cells, alongside CXCR4. Since maraviroc selectively inhibits CCR5, CXCR4-mediated signaling remains functional. Consequently, T cell–dependent B cell activation can proceed unimpaired (39).

### 4.3 Role of brown adipose tissue in orbit and the effect of maraviroc

One of the primary features of TED is the dysregulation of orbital adipose tissue, which contributes to clinical manifestations such as proptosis (eye bulging) and lid retraction. Morphological changes in orbital adipose tissue, such as smaller fat vacuoles (29). have been also observed in human fat (40). Orbital adipose tissue has distinct characteristics compared to other body adipose tissues, with orbital fibroblasts exhibiting mesenchymal stem cell (MSC) traits that allow them to differentiate into adipocytes under certain conditions (10, 41–43). In TED, activation of the TSHR in orbital fibroblasts promotes adipogenesis and hyaluronic acid production. Additionally, hyperthyroidism can stimulate both brown adipose tissue and brite (brown-in-white) adipose tissue (44). Previous studies have shown increased markers for white adipose tissue and brite in TED patients, particularly those with GD (44, 45).

In an animal experiment, high levels of skeletal muscle-specific CCL5 promote the migration of subcutaneous adipocytes into skeletal muscle, increasing intramuscular fat content. The application of the CCL5/CCR5 inhibitor, maraviroc, reduced this migration (46). A similar effect could explain the changes observed in our study. In another study, maraviroc treatment significantly decreased high-fat diet-induced body weight gain and reversed the high fat diet-suppressed expression of Glucose Transporter Type 4 and Insulin Receptor Substrate-1in adipose tissue (47).

Importantly, our *in vitro* experiments demonstrate that maraviroc directly suppresses adipogenic differentiation and CCL5 secretion in OF. Similar findings have been reported in other adipogenic cell systems. In 3T3-F442A preadipocytes, CCL5 was shown to promote adipocyte differentiation through CCR5 signaling, while maraviroc significantly attenuated this process, indicating that CCR5 directly contributes to adipogenic programming (48). Likewise, in a high-fat diet mouse model, maraviroc reduced adipose tissue macrophage recruitment and improved metabolic parameters, further supporting a role for CCR5 blockade in limiting pathological adipose tissue remodeling (49, 50).

The observed Reduction of adipogenesis in OF and the normalization of brown adipose tissue in TSHR-immunized mice treated with maraviroc (**Figures 5A, B**), suggests that maraviroc could be a therapeutic option for proptosis reduction. However, the question remains whether this is only a preventive measure or if maraviroc can even induce lipolysis once adipogenesis has already occurred. This question will need to be addressed in a follow-up animal experiment at a later stage of disease progression.

### 4.4 Maraviroc alters orbital lipid metabolism

Untargeted lipidomic profiling via UHPLC-QTOF analysis revealed clear distinctions between untreated and maraviroc-treated mice. Partial Least Squares Discriminant Analysis (**Figure 9A**) and heatmap clustering (**Figure 9B**) indicated significant remodeling of the orbital lipidome.

Lipidomic analysis revealed clear differences between TSHR- and TSHR-maraviroc-treated mice, indicating that maraviroc modulates orbital lipid metabolism. The heatmap (**Figure 9B**) shows distinct and consistent shifts in lipid expression while the volcano plot (**Figures 10A, B**) highlights significant changes in triacylglycerols (TGs), carnitines, sterols, and phosphatidylcholine. In total,25 lipids were significantly altered, characterized by reduced TGs and increased carnitines in the maraviroc-treated group, indicating a shift in lipid metabolism.

These changes may indicate a shift away from lipid storage toward increased fatty acid utilization in the orbital tissue. The reduction in brown adipose tissue further supports this interpretation, indicating decreased lipid accumulation and thermogenic activity in the orbit.

Mechanistically, the observed lipid shifts may relate to CCR5 inhibition by maraviroc. A recent study in 3T3-F442A preadipocytes demonstrated that CCL5-CCR5 signaling promotes adipogenic differentiation, whereas maraviroc suppresses this process (48). This aligns with our findings of reduced triacylglycerols and increased carnitines, which may indicate decreased lipid storage and altered fatty acid handling in orbital tissue. Together with our *in vitro* data showing that maraviroc inhibits adipogenesis in primary orbital fibroblasts, these results support a role for CCR5 blockade in modulating adipogenic and metabolic pathways. In addition to the changes in triacylglycerols and carnitines, we also observed alterations in sterol and phosphatidylcholine levels. Sterols and phospatidylcholines are key, membrane components involved in regulating inflammatory signaling and adipogenic pathways, and their modulation may reflect shifts in membrane dynamics and cellular activation states in OF. Such changes could influence both inflammation and adipogenesis in TED.

Maraviroc has been reported to affect lipid metabolism in various studies. In a mouse model of non-alcoholic fatty liver disease, maraviroc reduced hepatic triglyceride content and downregulated lipogenic and insulin resistance-associated genes, while increasing expression of ß-oxidation-related genes such as CPT1A. These findings support the possibility that CCR5 inhibition can modulate lipid metabolic pathways, consistent with the lipidomic changes observed in our study (50) (51).

These lipidomic changes are relevant in the context of GD and TED, where dyslipidemia is increasingly recognized. Patients often show elevated total cholesterol and LDL levels, and higher cholesterol has been associated with greater TED severity (52–54). Such systemic lipid abnormalities may contribute to orbital fat expansion and inflammation. In the setting, the ability of maraviroc to shift lipid metabolism, reflected by reduced triacylglycerols, increased carnitines, and changes in sterol and phosphatidylcholine pathways, may help counteract metabolic conditions that promote orbital tissue remodeling.

### 4.5 Immune cell infiltration and maraviroc’s impact on inflammation

A central mechanism of TED pathogenesis is immune cell infiltration and inflammatory processes, particularly the recruitment of T-cells and macrophages to the orbit. Our study found that maraviroc treatment significantly reduced CCL5-positive cells (**Figures 5E, F**), which are key players in the inflammatory response. CCL5, a pro-inflammatory chemokine, recruits immune cells like T-cells and macrophages to sites of inflammation, contributing to the chronic inflammation characteristic of TED. Moreover, hypoxia is another characteristic feature of GO, where we have earlier shown the hypoxia marker HIF-1alpha together with TNFα leads to increased expression of CCL5 in the orbital tissue (55). This in combination with other chemokines such as CXCL9, CXCL10 and CXCL11 may lead to a change in the balance between TH1 and TH2 subpopulations of CD4 T cells in the orbit for disease progression to be manifested (56). The reduction of CCL5-positive cells in maraviroc-treated mice suggests that CCR5 blockade may limit inflammatory cell recruitment to the orbit. Our *in vitro* experiments further support this mechanism: IL-1β-stimulated orbital fibroblasts produced high levels of CCL5, which were markedly reduced by maraviroc. These findings indicate that fibroblasts represent an important local source of CCL5 and that their chemokine output is sensitive to CCR5 inhibition.

Together, these results support a model in which both stromal and immune cells contribute to CCL5-mediated CCR5 signaling in the orbit, and in which maraviroc interferes with this pathway at multiple levels, thereby attenuating inflammatory amplification in TED. While maraviroc treatment did not significantly affect TNFα-dependent macrophage infiltration (**Figures 6A, B**), it reduced F4/80^+^ macrophage infiltration, indicating that maraviroc selectively modulates macrophage recruitment or activation. Total CD3^+^ T-cell numbers showed only a modest decrease; however, subset analysis revealed a clear reduction in CD4^+^ T cells, whereas CD8^+^ T cells were largely unaffected. This pattern is consistent with the higher CCR5 expression reported on activated CD4^+^ T cells compared with CD8^+^ T cells (57), providing a plausible explanation for their differential response to maraviroc. This selective modulation of immune cell recruitment suggests that maraviroc could help mitigate the inflammatory and fibrotic processes in TED by targeting both lipid metabolism and immune cell recruitment and responses.

Maraviroc, as a selective CCR5 antagonist, modulates immune cell infiltration by interfering with chemokine-driven recruitment of leukocytes into inflamed tissues. CCR5 is highly expressed on monocytes, macrophages, and T cells, and its ligands, particularly CCL5 (RANTES), play a key role in trafficking these immune cells to sites of inflammation.

In high-fat diet models, maraviroc has been shown to significantly reduce macrophage accumulation in visceral adipose tissue, accompanied by lower levels of inflammatory cytokines such as TNFα and IL-6 (49, 58). This is achieved through downregulation of MCP-1 expression and inhibition of JNK and NF-κB signaling, key pathways in obesity-induced inflammation (50). Furthermore, CCR5 deficiency reduces macrophage accumulation in adipose tissue and promotes a shift from the proinflammatory M1 phenotype toward the anti-inflammatory M2 phenotype, which is associated with improved insulin sensitivity and decreased tissue inflammation in obesity models. In adipose tissue and other inflamed environments, CCR5 blockade also limits CD4^+^ and CD8^+^ T-cell infiltration, reducing the overall pro-inflammatory immune milieu (36).

Together, these findings indicate that maraviroc attenuates key inflammatory processes in TED by reducing the recruitment of immune cells, particularly macrophages and CD4^+^ T cells. This immunomodulatory activity complements the metabolic effects observed in orbital tissue, suggesting that maraviroc may counteract both inflammatory and adipogenic pathways that drive disease progression.

## 5 Conclusion

In conclusion, our study demonstrates that maraviroc effectively improves disease outcome in a murine model of GD, primarily by preventing orbital remodeling and limiting disease progression Although its effects on autoimmune hyperthyroidism were modest, maraviroc showed clear therapeutic activity within the orbit. It reduced the expansion of brown adipose tissue in TSHR-immunized mice and inhibited adipogenic differentiation of primary orbital fibroblasts, highlighting a direct anti-adipogenic effect. Lipidomic analyses further revealed distinct alterations in lipid metabolism, including reduced triacylglycerols and increased carnitines, as well as changes in sterol and phosphatidylcholine pathways, indicating a broader impact on metabolic processes associated with orbital fat accumulation. Maraviroc also decreased CCL5-positive cells and reduced infiltration of F4/80^+^ macrophages and CD4^+^ T cells, supporting a key role for the CCR5-CCL5 axis in driving inflammatory amplification in TED. Our additional experiments confirmed that orbital fibroblasts themselves are an active source of CCL5 and that their chemokine secretion is sensitive to CCR5 inhibition.

Importantly, maraviroc’s dual anti-adipogenic and anti-inflammatory effects directly target two central pathogenic mechanisms of TED, offering a comprehensive therapeutic approach with a favorable safety profile and potentially fewer side effects compared to current immunosuppressive treatments. Despite its limited influence on thyroid function, these findings support further exploration of maraviroc as a promising treatment strategy for TED. Further studies, particularly in human subjects, are warranted to confirm these results and to assess the long-term efficacy and safety of CCR5 antagonism in the clinical management of TED.

## Data Availability

The raw data supporting the conclusion of this article will be made available by the authors, without undue reservation.
