# Supplementary material for: Maraviroc attenuates orbital remodeling, inflammation, and lipid dysregulation in a murine model of thyroid eye disease associated with Graves’ disease
Source: Front Endocrinol (Lausanne). 2026 Feb 27;17:1717212. doi: 10.3389/fendo.2026.1717212 (PMC12982029; doi:10.3389/fendo.2026.1717212)
Supplement: Supplementary file 1 [file DataSheet1.docx]

Supplementary Material

## Supplementary Figures

**Supplementary Fig. 1 Identification of CCL5-Producing Cell Types in Orbital Tissue and Orbital Fibroblasts.** (A) Representative immunofluorescence co-localization images of orbital sections from TSHR-immunized mice showing CCL5 expression together with cell lineage markers. CCL5⁺ cells co-express vimentin (fibroblasts), F4/80 (macrophages), and CD3 (T cells), indicating that multiple stromal and immune cell populations contribute to CCL5 production in the orbit during disease. Nuclei were counterstained with DAPI. Scale bars: 20 µm. (B) CCL5 secretion from orbital fibroblasts isolated from orbital tissue samples. Fibroblasts were stimulated with IL-1β to induce CCL5 production and subsequently treated with maraviroc. Maraviroc markedly reduced CCL5 secretion, approaching baseline levels, demonstrating that orbital fibroblasts are an active source of CCL5 and that their chemokine production is sensitive to CCR5 pathway inhibition. Data are shown as mean ± SD (n = 3). Statistical significance was assessed by one-way ANOVA; p < 0.05 (*), p < 0.001 (**), p < 0.001 (***), p < 0.0001 (****).

**Supplementary Fig. 2 In vitro Adipogenesis of Orbital Fibroblasts.** Orbital fibroblasts were isolated from orbital tissue samples and subjected to adipogenic differentiation assays. (A) Representative images of lipid droplet formation after fixation and Nile Red-FITC staining are shown (10× magnification). (B) Nile Red fluorescence intensity was measured using an ELISA reader to quantify lipid accumulation relative to the control. Data are presented as mean ± SD. Statistical significance was determined using a paired Student’s t-test.

**Supplementary Fig. 3 Effect of Maraviroc on TNF-α Expression in Orbital Tissue**. Orbital tissues were collected, fixed, and paraffin-embedded. Serial mid-orbital sections were stained with antibodies against TNF-α. Quantification of TNF-α⁺ cells across the entire orbital section are shown. Data are presented as mean ± SD (n = 12 per group: β-Gal, TSHR, and TSHR + maraviroc). Statistical significance was determined using one-way ANOVA (*p < 0.05; **p < 0.01).
